# Clinical, Virological and Immunological Features from Patients Infected with Re-Emergent Avian-Origin Human H7N9 Influenza Disease of Varying Severity in Guangdong Province

**DOI:** 10.1371/journal.pone.0117846

**Published:** 2015-02-27

**Authors:** Zi Feng Yang, Chris Ka Pun Mok, Xiao Qing Liu, Xiao Bo Li, Jian Feng He, Wen Da Guan, Yong Hao Xu, Wei Qi Pan, Li Yan Chen, Yong Ping Lin, Shi Guan Wu, Si Hua Pan, Ji Cheng Huang, Guo Yun Ding, Kui Zheng, Chang Wen Ke, Jin Yan Lin, Yong Hui Zhang, Horace Hok Yeung Lee, Wen Kuan Liu, Chun Guang Yang, Rong Zhou, Joseph Sriyal Malik Peiris, Yi Min Li, Rong Chang Chen, Ling Chen, Nan Shan Zhong

**Affiliations:** 1 State Key Laboratory of Respiratory Disease, National Clinical Research Center for Respiratory Disease, First Affiliated Hospital of Guangzhou Medical University, Guangzhou, Guangdong, China; 2 Health quarantine (BSL-3) Lab, Guangdong Inspection and Quarantine Technology Center, Guangzhou, China; 3 Guangdong Center for Disease Control and Prevention, Guangzhou, China; 4 Centre of Influenza Research, School of Public Health, HKU Li Ka Shing Faculty of Medicine, The University of Hong Kong, Hong Kong; 5 HKU-Pasteur Research Pole, School of Public Health, HKU Li Ka Shing Faculty of Medicine, The University of Hong Kong, Hong Kong; University Hospital San Giovanni Battista di Torino, ITALY

## Abstract

**Background:**

The second wave of avian influenza H7N9 virus outbreak in humans spread to the Guangdong province of China by August of 2013 and this virus is now endemic in poultry in this region.

**Methods:**

Five patients with H7N9 virus infection admitted to our hospital during August 2013 to February 2014 were intensively investigated. Viral load in the respiratory tract was determined by quantitative polymerase chain reaction (Q-PCR) and cytokine levels were measured by bead-based flow cytometery.

**Results:**

Four patients survived and one died. Viral load in different clinical specimens was correlated with cytokine levels in plasma and broncho-alveolar fluid (BALF), therapeutic modalities used and clinical outcome. Intravenous zanamivir appeared to be better than peramivir as salvage therapy in patients who failed to respond to oseltamivir. Higher and more prolonged viral load was found in the sputum or endotracheal aspirates compared to throat swabs. Upregulation of proinflammatory cytokines IP-10, MCP-1, MIG, MIP-1α/β, IL-1β and IL-8 was found in the plasma and BALF samples. The levels of cytokines in the plasma and viral load were correlated with disease severity. Reactivation of herpes simplex virus type 1(HSV-1) was found in three out of five patients (60%).

**Conclusion:**

Expectorated sputum or endotracheal aspirate specimens are preferable to throat swabs for detecting and monitoring H7N9 virus. Severity of the disease was correlated to the viral load in the respiratory tract as well as the extents of cytokinemia. Reactivation of HSV-1 may contribute to clinical outcome.

## Introduction

A novel avian H7N9 influenza A virus emerged in March of 2013 causing serious human disease and death in China [1,2]. Up to 5 May 2014, 432 confirmed human cases leading to more than 160 deaths have been reported (http://www.cnic.org.cn/uploadfile/2014/0513/20140513031342659.pdf). While the initial outbreak of the H7N9 in humans occurred around the Yangtze River delta in March and April 2013, the infection spread in poultry to South China and the second wave of the epidemic affected South of China including Guangdong and Hong Kong, in the winter of 2013 [3,4]. During the first wave of outbreak, the majority of the reported cases of A/H7N9 disease were patients with fulminant viral pneumonia, identified through the national surveillance system for pneumonia of unknown etiology [5]. In the reported case series, A/H7N9 patients typically developed a rapidly progressive viral pneumonia leading to respiratory failure and acute respiratory distress syndrome (ARDS) reminiscent of human HPAI H5N1 disease [2,3,6,7].

There are now over 102 A/H7N9 cases reported within Guangdong Provence since August of 2013 suggesting that this region is now endemic for A/H7N9 virus. The numbers of new cases appear to be increasing recently. Cases identified in Hong Kong were also probably acquired infection through contact with live poultry in Guangdong Province [4]. Detailed study on the viral loads, cytokines and clinical outcome on patients infected by the re-emergent H7N9 strain is still lacking.

In this study, we summarized the clinical manifestations and disease progression of five patients who were infected with the H7N9 viruses in Guangdong Province. We correlated their disease progression and clinical outcome with viral load and cytokine levels in plasma. The effect of neuraminidase inhibitors and convalescent plasma therapy was also investigated.

## Materials and Methods

### Patients

Five consecutive patients diagnosed as laboratory-confirmed avian influenza A (H7N9) virus infection at the First Affiliated Hospital of Guangzhou Medical University were included in this study. All patients initially presented at local primary health care clinics or local hospitals before they were referred to the Intensive Care Unit (ICU) of the First Affiliated Hospital of Guangzhou Medical University. At the time of enrolment and hospitalization, the subject’s clinical history, physical examination, radiological findings, hematological, biochemical and microbiological investigations were recorded. We defined acute respiratory distress syndrome on the basis of the Berlin Definition [8]. Presumed incubation period was defined as the time between last poultry exposure and the onset of symptoms. Approval for the study was obtained from the ethics committee of the First Affiliated Hospital of Guangzhou Medical University and written informed consent was obtained from the patients or their family members

### Detection of virus infection and viral load

Throat swabs, conjunctival swab, bronchoalveolar lavage fluid (BALF), sputum, endotracheal aspirate, urine, and/or fecal samples were collected from the day of admission to ICU throughout the period of hospitalization. The viral RNA from the samples was extracted using QIAamp MinElute Virus Spin kit (Qiagen, Valencia, USA) according to the manufacturer’s instructions. Avian influenza A(H7N9) virus was detected using the avian influenza A virus H7N9 Real Time RT-PCR kit (Shanghai ZJ Bio-tech Co., Ltd., Shanghai, China) which has approved by the Chinese Food and Drugs Agency. Viral RNA concentration was quantified by real-time PCR in parallel with standards with a known copy numbers of the haemagglutinin gene cloned in a plasmid. Viral load was inferred as copy numbers per ml of samples in the lysis buffer. Viral DNA was extracted by the QIAamp DNA Mini Kit (Qiagen) for detection of HSV-1 by PCR and the viral load was inferred as ΔCT value.

### Detection of cytokine levels

Cytokines (IL-8, IP-10, interferon-α, MIP-1α, MIP-1β, MCP-1, MIG, IL-1β) from the BALF and plasma samples of the patients was determined using a CBA human inflammatory cytokine kit (BD Biosciences, San Jose, CA, USA) according to the manufacturer’s instructions. Data acquisition was performed on BD LSR Fortessa (BD Biosciences). Data were analyzed by CBA analysis software.

### Serology test

Titers of anti-H7N9 influenza antibody were measured by hemagglutination inhibition (HI) assay and microneutralization-ELISA (micro NT-ELISA) assays according to the WHO protocols [9, 10]. Horse red blood cells were used in the HI assay. A/Anhui/01/2013 H7N9 virus was used as the reference antigen or virus for the serology tests. All bioassays were conducted in a BSL-3 laboratory.

## Results

The first five patients admitted to our intensive care unit, from August 2013 to February 2014, were included in this study and this includes the first human case of H7N9 infection in Guangdong province (Patient 1). The age of the patients ranged from 39 to 66 years. Four of the patients survived and were discharged from hospital finally while one patient (Patient 3) died. Four of them had exposure history to live poultry within 14 days of onset of clinical symptoms. The demographic characteristics are shown in supplementary table 1 (S1 Table) and the radiological findings at the day of admission are shown in supplementary figure 2 (S2 Fig.).

Fever ≥38.5°C, fatigue, cough, sputum production and shortness of breath were reported by the five patients at the onset of illness but there was no evidence of conjunctivitis (Table 1) and virus was not detected in any of the conjunctival swabs tested. Once they were confirmed to have H7N9 infection, all the patients were commenced on 150 mg of oseltamivir twice daily. Meropenem (1g, bid), Vancomycin (500mg, tid) or Tienam (1g tid) were generally used as the first line of antibiotic treatment according to community-acquired pneumonia guidelines [11]. Antiviral therapy was altered in the event of non-response to the initial antiviral regimen. The type and the dosages of the drugs given to patients 1 to 5 during hospitalization is summarized in supplementary table 2 (S2 Table).

**Table 1:** Clinical symptoms, complications, treatment, and clinical Outcomes.

	Patient 1	Patient 2	Patient 3	Patient 4	Patient 5
**APACHE II score**	10	15	19	10	3
**Clinical symptoms**					
**Fever**	Yes	Yes	Yes	Yes	Yes
**Maximal temperature (℃)**	39.5	38.7	39.4	40.5	39.9
**Fatigue**	Yes	Yes	Yes	Yes	Yes
**Cough**	Yes	Yes	Yes	Yes	Yes
**Sputum production**	Yes	Yes	Yes	Yes	Yes
**Hemoptysis**	No	No	No	Yes	No
**Shortness of breath**	Yes	Yes	Yes	Yes	Yes
**Diarrhea or vomiting**	Yes	No	No	No	No
**Conjunctivitis**	No	No	No	No	No
**Complications**					
**Pneumonia**	Yes	Yes	Yes	Yes	Yes
**Acute respiratory distress syndrome**	Yes	Yes	Yes	Yes	Yes
**Shock**	No	Yes	Yes	No	No
**Acute kidney injury**	No	No	Yes	No	No
**Rhabdomyolysis**	No	No	No	No	No
**Bacteria isolation from culture**	No	No	No	No	No
**Treatment**					
**Antiviral drugs**	Yes	Yes	Yes	Yes	Yes
**Oxygen therapy**	Yes	Yes	Yes	Yes	Yes
**Extracorporealmembrane oxygenation**	No	No	Yes	No	No
**Mechanical ventilation**	Invasive	Invasive	Invasive	Noninvasive	Noninvasive
**Continuous renal-replacement therapy**	No	No	Yes	No	No
**Antibiotics**	Yes	Yes	Yes	Yes	Yes
**Antifungal drugs**	Yes	Yes	Yes	Yes	No
**Glucocorticoids**	Yes	Yes	Yes	No	No
**Intravenous immune globulin**	Yes	Yes	Yes	No	No
**Clinical outcome**	Discharged	Discharged	Death	Discharged	Discharged

**Fig 1:**
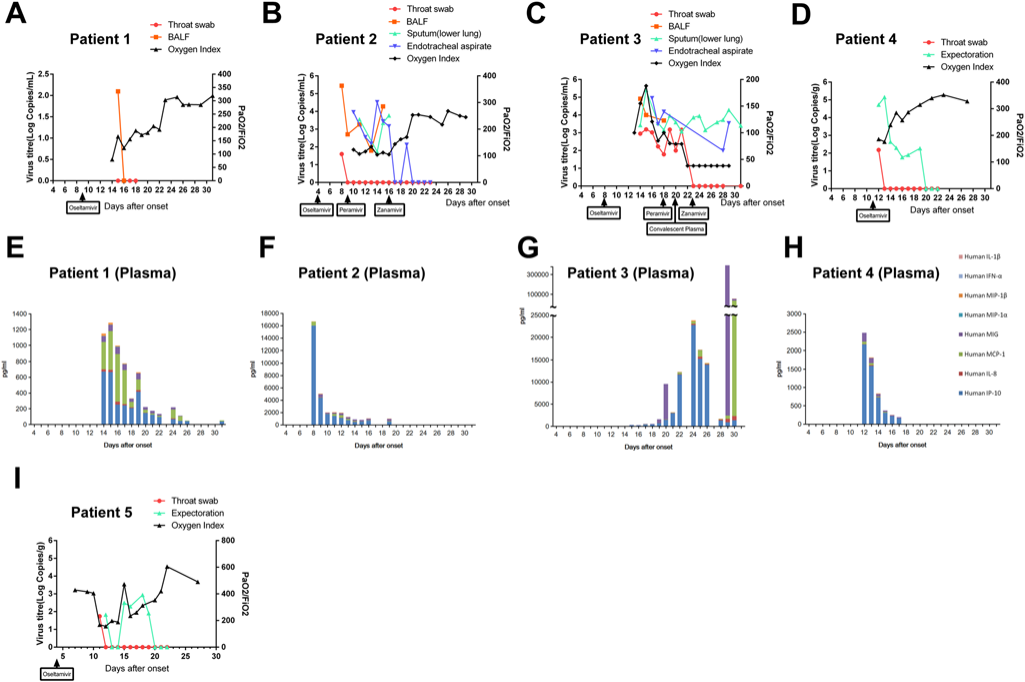
Viral load and oxygen index in different clinical specimens, (A-D, I) and plasma cytokine levels (F-H) in H7N9-infected patients. Patient 1 (A, E); Patient 2 (B, F); Patient 3 (C, G); Patient 4 (D, H); Patient 5 (I). The commencement of antiviral drugs oseltamivir, and where relevant, peramivir, intravenous zanamivir or convalescent plasma is indicated in Figs. A-D, I).

Patient 1 had pneumonia and acute respiratory distress syndrome (ARDS) at admission to the ICU (day 9 after disease onset). Her APACHEII score was 10 at the first day in ICU. Commencement of oseltamivir treatment was associated with a fall of viral load from the throat and BALF samples, turning negative at day 16 post illness and was associated with increasing of oxygen index (Fig. 1A). Decreasing levels of pro-inflammatory cytokines in plasma were observed (Fig. 1E).

Both patient 2 and 3 developed ARDS accompanied by shock. Their APACHEII score were 15 and 19 respectively at the first day in ICU. High total leukocyte and neutrophils counts were found in peripheral blood suggesting that secondary bacterial infection had already occurred (S3 Table). Additional antibiotics were therefore given (S2 Table). While the leukocyte counts returned to normal ranges in patient 2 after 20 days of the disease onset, leukocyte counts in patient 3 remained elevated throughout hospitalization. However, no bacteria could be cultured from the sputum or BALF samples in either patient. Their fecal specimens and plasma was also collected at these time points but were all negative for H7N9 RNA by RT-PCR. The viral load remained at 10^5^ to 10^4^ copies per ml in BALF specimens although both patients had been given oseltamivir for three to five days (Fig. 1B and C). No oseltamivir resistant mutations (neuraminidase gene R292K) were found from the H7N9 NA gene amplified by PCR (data not shown). Changing the antiviral drug to peramivir did not suppress H7N9 viral load but subsequent change to intravenous zanamivir was associated with a rapid decrease in the viral load in patient 2 (Fig. 1B). This was also associated with progressive improvement in the oxygen index. HSV-1 DNA (CT value 35.02) was identified from the throat specimen of patient 2 at day 23 after the disease onset (Table 2). IP-10 was the predominant plasma cytokine detected in the early days post admission (Fig. 1F). Interestingly, concentrations of MIG were much higher in the BALF than in plasma while IL-8 was only detectable in the BALF (Table 3).

**Table 2:** Detection of HSV-1 from the patients.

Patient 2		Patient 3		Patient 4	
Days of disease onset	CT value (type of specimen)	Days of disease onset	CT value (type of specimen)	Days of disease onset	CT value (type of specimen)
23	HSV-35.02 (throat swab)	23	HSV-30.63 (throat swab)	13	HSV-33.67 (throat swab);
		24	HSV-26.85 (throat swab)	16	HSV-31.73 (ETA)
		25	HSV-33.47 (throat swab)		
		26	HSV-24.15 (throat swab)		
		27	HSV-23.07 (throat swab)		
		28	HSV-24.73 (throat swab)		
		29	HSV-18.05 (throat swab)		
		31	HSV-17.74 (throat swab); HSV-30.35 (sputum from lower lung); HSV-32.02 (plasma)		

**Table 3:** Comparison of the cytokine levels (pg/ml) from plasma and BAL.

Days after onset	8		9		11		13		15	
Patient 2	Plasma	BALF	Plasma	BALF	Plasma	BALF	Plasma	BALF	Plasma	BALF
IP-10	15970.4	4302.5	4402.4	958.5	1407.3	61.8	732.1	3058.3	497.7	1097.8
IL-8	30.0	153.8	17.3	733.6	26.4	852.0	16.5	2485.4	11.3	958.7
MCP-1	606.0	0.0	55.9	320.9	325.9	0.0	206.8	6522.5	45.2	44.7
MIG	0.0	7859.8	473.8	92.5	186.8	30.7	269.7	591.5	227.4	1046.2
MIP-12	2.8	35.6	1.6	0.0	1.4	0.0	1.3	0.0	0.0	13.0
MIP-12	62.6	0.0	57.3	14.2	71.0	0.0	24.2	43.7	2.5	14.7
IFN-	9.1	23.0	0.0	0.0	0.0	0.0	0.0	0.0	0.0	0.0
IL-1 2	0.0	0.0	0.0	0.0	0.0	48.4	0.0	0.0	0.0	0.0

Days after onset	15		16		18					
Patient 3	Plasma	BALF	Plasma	BALF	Plasma	BALF				
IP-10	217.8	110.7	179.4	644.0	400.7	832.2				
IL-8	0.0	5275.5	0.0	5404.4	0.0	29.3				
MCP-1	0.0	40.6	1.9	41.1	17.3	0.0				
MIG	53.3	306.7	48.6	1253.2	114.4	339.1				
MIP-1	0.0	21.9	0.0	154.9	0.0	11.8				
MIP-1	0.0	29.0	0.0	22.4	0.0	0.0				
IFN-	0.0	0.0	0.0	0.0	0.0	0.0				
IL-1	0.0	185.2	0.0	394.4	0.0	12.5				

In patient 3, neither oseltamivir, peramivir nor intravenous zanamivir was associated with suppression of H7N9 viral load in the sputum of endotracheal aspirate (around 10^3^ to 10^4^ viral copies per ml) although the throat swab became negative on day 23 post disease onset. Because of his adverse clinical condition and lack of response to all three antiviral drugs, one dose of convalescent plasma which had been collected from a recovered H7N9 patient was injected intravenously at day 20 after his disease onset. There was no improvement in the oxygen index even after the commencement of intravenous immune globulin (Fig. 1C). This patient already had H7N9 antibody titers of 1:320 by the HI test and 1:160 by the microneutraliation test prior to the therapy with convalescent plasma. There was an increase in neutralizing antibody titer to H7N9 was detected in his serum immediately after the injection of convalescent plasma (S1 Fig.). The plasma levels of IP-10 remained elevated to levels higher than seen in patients 1 or 2 through day 22–26 of illness and there was a further dramatic increase of plasma MIG and MCP-1 on day 29–30 (Fig. 1G). There were very high levels of IL-8 in BALF that was not detected in plasma (Table 3). The patient died from sepsis with multi-organ failure at day 32 after disease onset [12]. Importantly, he had detectable HSV DNA in the throat swabs from day 23 post onset (CT 30.63) and the viral load progressively increased (CT 18.05) by day 29 of illness. At this time (day 31 post onset) HSV DNA was also detected in sputum (CT value 30.35) and plasma (CT value 32.02) (Table 2). These HSV PCR results were only available in retrospect and these patients were not treated with acyclovir.

Patient 4 and 5 were less severely ill at presentation with an oxygen index of around 150 to 400. Their APACHEII scores were 10 and 3 respectively, on the first day in ICU. No intubation was required but only non-invasive nasal mask ventilation was given during hospitalization. They were commenced on oseltamivir on day 11 (Patient 4) and day 4 (Patient 5) of illness, respectively. H7N9 RNA detection from their throat swabs was positive only for one day after hospitalization (day 12 and day 11 of disease onset, respectively). However, virus remained detectable in their sputum specimens for much longer until day 20 of illness. Faecal and plasma specimens remained negative for viral RNA. Increasing oxygen index was seen following the commencement of oseltamivir (Fig. 1D and I). Pro-inflammatory cytokines in plasma of patient 4 were also not very high and gradually declined with the reducing viral load (Fig. 1H). Reactivation of HSV was identified from the throat and ETA samples of patient 4 on day 13 and 16 after disease onset (Table 2).

## Discussion

We compared and summarized the serial H7N9 viral load in throat swab, sputum, endotracheal aspirates and BALF; with plasma and (in patients 2 and 3) BALF cytokine levels and with oxygen index and clinical outcome of patients with differing clinical severity. These data have been correlated with antiviral (oseltamivir, peramivir and intravenous zanamivir) therapy and in one patient with the effects of intravenous therapy with convalescent plasma. The serial viral neutralization and HI antibody titers are also reported. All patients had a history of exposure to poultry suggesting that these may be zoonotic infections from poultry. The initial clinical symptoms of the patients were similar to that previously reported with fever, fatigue, cough, sputum production and shortness of breath being the common early clinical features [6]. Viral load detected from sputum, which can be obtained from non-intubated patients, provided comparable sensitivity to the BALF or ETA and was superior to throat swabs. The serial viral load data shows that clearance of virus from throat swab specimens is not a reliable index therapeutic success. This was most clearly seen with patients 2 and 3 where virus in throat swabs became undetectable although virus remained at high titer in deep respiratory specimens (sputum, endotracheal aspirate, BALF), indicating lack of virological response which correlated with clinical outcome. In patient 2, throat swab viral load falsely suggested response to oseltamivir or peramivir therapy although deep respiratory specimens endotracheal aspirate, sputum, BALF) did not. The viral load in deep respiratory specimens correlated better with the oxygen index, showing that intravenous zanamivir led to virus clearance and clinical improvement. In patient 3, the throat swab viral load again falsely suggested H7N9 virus clearance associated with antiviral therapy although virus continued to be detected in sputum and endotracheal aspirates with poor oxygenation and this patient had a fatal outcome.

The antiviral effect of oseltamivir varies among the patients although it is still our front line anti-viral drug given to the patients with H7N9 infection. We observed that oseltamivir therapy was associated falling viral load and clinical response in patients 1, 4 and 5 even though they were commenced on therapy in the second week of illness. By the time oseltamivir therapy was commenced, these three patients were also mounting neutralizing and HI antibody responses to H7N9. Thus it is not clear whether they were responding to the antiviral therapy or were clearing the virus via their own adaptive immune responses. Peramivir did not appear to be effective salvage therapy for patients 2 and 3 who failed to respond virologically and clinically to oseltamivir. However, intravenous zanamivir was associated with virological and clinical response in patient 2 but not in patient 3. Autopsy results showed that the pulmonary alveoli were partially collapsed in patient 3, with atypical hyperplasia and greater prominence of nuclei in pulmonary alveolar epithelial cell (unpublished data). The presence of squamous metaplasia of the bronchial epithelium, interstitial fibrous tissue hyperplasia and lymphocyte infiltration were identified in lung. Such pathological changes were in accordance with interstitial pneumonia accompanied by alveolar epithelial atypical adenomatous hyperplasia. Taken the factors together including pathologic changes, long persistence of viral shedding, and HSV infection, immune-suppression may exist and contribute to severe disease outcome of patient 3. However, whether this was the reason that patient 3 was irresponsive to the antiviral treatment is not clear.

It has been reported that treatment of severe H1N1 2009 infection with convalescent plasma reduced respiratory tract viral load, serum cytokine response, and mortality [13]. However, in patient 3, one dose of convalescent plasma from a patient who had recovered from H7N9 disease failed to reduce viral load in the sputum or endotracheal aspirate and had no beneficial clinical effect on oxygenation. By the time that convalescent plasma was administered to this patient (day 20 of illness), there was already a good HI and neutralizing antibody response. Although the plasma infusion was associated with a transient increase in the neutralizing antibody response, it may have been too late in the clinical course to effect clinical benefit. Another possibility is that no enough convalescent plasma for following use, leading to its clinical benefit unclear. Earlier intervention may have been more beneficial but the challenge is to identify those patients who will fail to respond to antiviral therapy early enough. Alternatively, it may be those patients who fail to make robust neutralizing antibody titers by day 10 of illness who may benefit most from passive antibody therapy [14]

Secondary bacterial infection is known to be an important cause of poor clinical outcome in influenza, including that caused by the pandemic H1N1 virus or H7N9 virus. [15–19]. In our therapeutic strategy, antibiotics were first given to the patients in the local hospitals according to community-acquired pneumonia guideline. In severe cases, additional antibiotics and anti-fungal therapy was administered. Although the prescribing of antibiotics may be associated with side effects and may increase antibiotic resistance, our primary aim is to reduce the risk of secondary infection in order to increase the patient survival rate. However, both patient 2 and 3 still showed evidence suggestive of secondary bacterial infection as reflected by their increasing white cell and neutrophil cell count in the blood. We suspect that bacterial infection may have existed before admission to our hospital.

Hypercytokinemia has been noted as a factor associated with adverse clinical outcome during influenza infection [19–21]. One study showed that the plasma levels of IL-6 and IP-10 in severe cases are higher than the non-severe cases [22], while another study found that IL-6, IL-8 and to a lesser extent, MIP-1β and IL-10 production was higher in fatal than the non-fatal cases [23]. Overall, we found the cytokine levels in plasma samples were substantially higher in the fatal case than the three survival cases (Fig. 1E to H) and the cytokine levels in the plasma samples of the survived patients declined along with the improvement of their condition while dramatic increase of cytokines were observed in patient 3 before he died. We found that fatal patient had much higher plasma levels of IL-8, IP-10, MCP-1, MIG and MIP-1β, and higher BALF levels of IL-1β and IL-8, compared to the survivors.

The levels of cytokines in the concurrent plasma and BALF samples showed remarkable differences. Some cytokines such as IL-8 were only elevated in BALF and high levels were noted in the BALF of the patient 3 (with fatal outcome) as early as day 15 of illness with moderate levels also seen in severely ill patient 2. Elevated level of IL-1β was only detectable in BALF. Levels of other chemokines such as IP-10 were as high or higher in the plasma compared to the BALF. Wang and colleagues also reported markedly different cytokine levels between plasma and BALF samples in fatal patients and that IL-8 was mainly detected in BALF samples [19]. Whether IL-8 in BALF is a maker associated secondary bacterial infection (which patients 2 and 3 probably had) or whether it is an independent marker of adverse clinical outcome deserves further study. Some cytokines such as IP-10, MCP-1 and MIG which have been associated to the pathogenicity of avian H7N9 influenza virus infection in humans [24] were not tested for in the study by Wang et al [23]. Our results compared the induction of these three cytokines in BALF vs. plasma between fatal and non-fatal patients. The levels of these cytokine in the BALF of fatal patient are generally higher than the corresponded plasma samples. The level of IP-10 and MCP-1 were higher in plasma than in BALF at the early disease onset of the non-fatal patient but an opposite trend was observed with the improvement of her condition.

In summary, our serial viral load, cytokine data and clinical outcome from 5 intensively investigated patients with diverse clinical outcome provide some insights for the monitoring and clinical management of patients with H7N9 disease. Deep respiratory specimens such as sputum or ETA specimens rather than throat swabs are crucial for monitoring the clinical response to therapy and likely for initial diagnosis as well. Long persistence of viral shedding in mild cases should attract people’s attention for progressing severe disease. Although our patient numbers are limited, peramivir does not appear to have any impact in salvage therapy of patients who fail to respond to oseltamvir, even in the absence of the NA R292K mutation while intravenous zanamivir appears to be more useful in this regard. A study is needed to prospectively compare the particular regimen was associated with superior outcomes or reduced emergence of resistance.

## Supporting Information

S1 Fig Results of hemagglutination inhibition (HI) and microneutralization (MN) antibody titres in H7N9 infected patients. (A) HI (B) MN.
(TIF)

S2 Fig Chest radiography of the H7N9-infected patients taken within two days of hospital admission. (A) Patient 1 (B) Patient 2 (C) Patient 3 (D) Patient 4.
(TIF)

S1 Table Demographic and Epidemiologic Characteristics. (DOCX)

S2 Table The summary of the drugs given to the patients. (DOCX)

S3 Table Laboratory examinatio (DOCX)
