# Supplementary figures and images for: Clinical, Virological and Immunological Features from Patients Infected with Re-Emergent Avian-Origin Human H7N9 Influenza Disease of Varying Severity in Guangdong Province
Source: PLoS One. 2015 Feb 27;10(2):e0117846. doi: 10.1371/journal.pone.0117846 (PMC4344233; doi:10.1371/journal.pone.0117846)

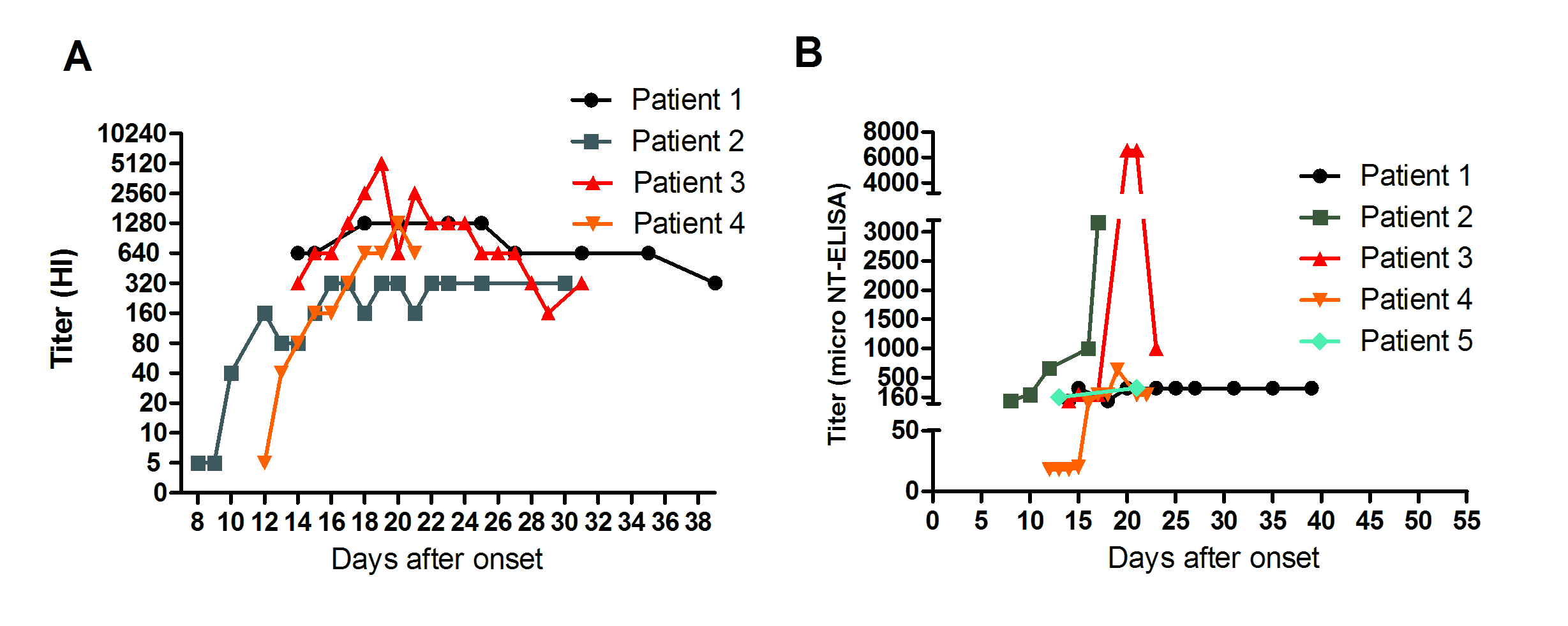

Supplement: S1 Fig — (A) HI (B) MN. (TIF) [file pone.0117846.s001.tif]

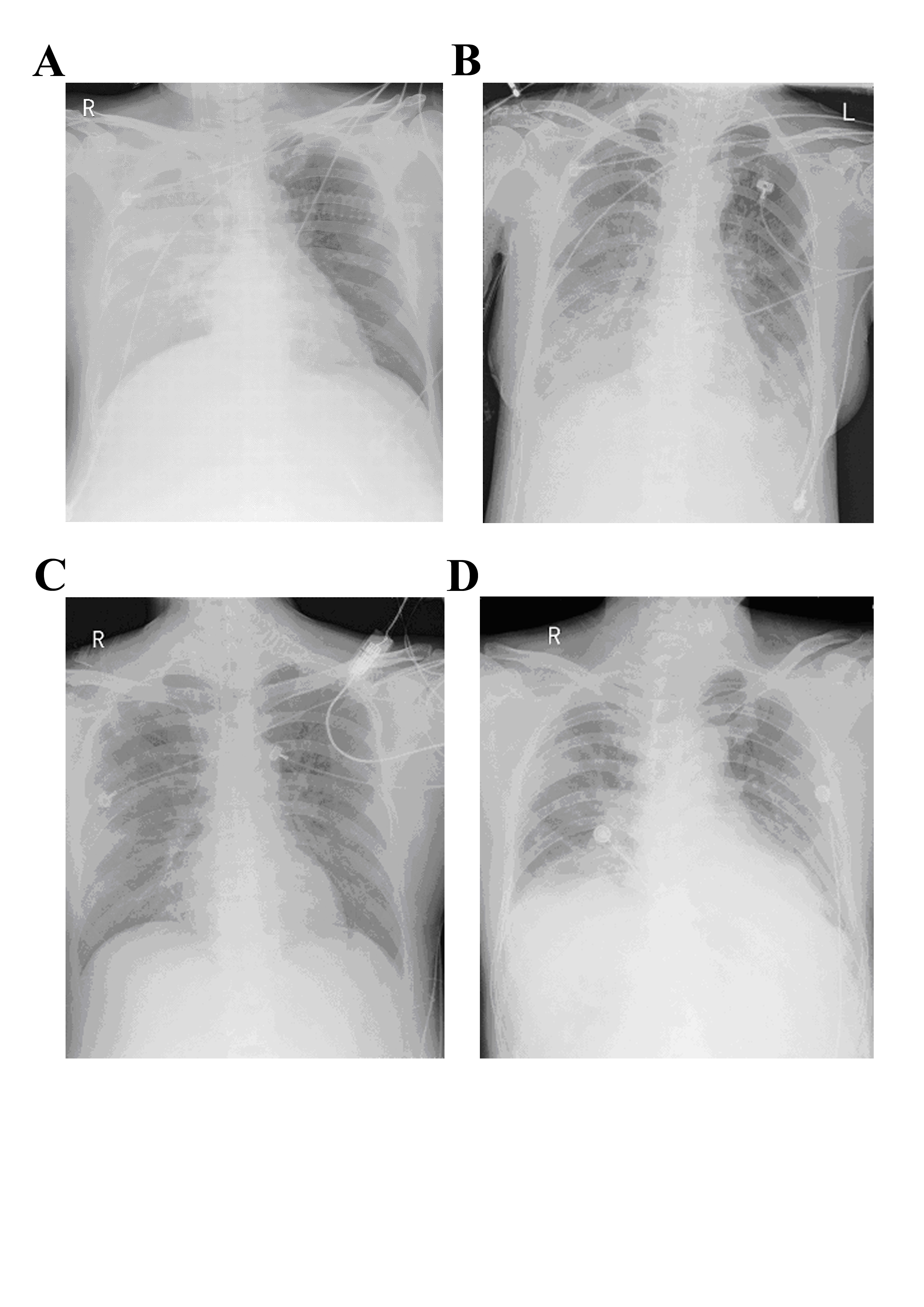

Supplement: S2 Fig — (A) Patient 1 (B) Patient 2 (C) Patient 3 (D) Patient 4. (TIF) [file pone.0117846.s002.tif]
