# Supplementary material for: Clinical, Virological and Immunological Features from Patients Infected with Re-Emergent Avian-Origin Human H7N9 Influenza Disease of Varying Severity in Guangdong Province
Source: PLoS One. 2015 Feb 27;10(2):e0117846. doi: 10.1371/journal.pone.0117846 (PMC4344233; doi:10.1371/journal.pone.0117846)
Supplement: S1 Table — (DOCX) [file pone.0117846.s003.docx]

S1 Table. Demographic and Epidemiologic Characteristics

| Characteristics | Patient 1 | Patient 2 | Patient 3 | Patient 4 | Patient 5 |
| --- | --- | --- | --- | --- | --- |
| Age—yr | 54 | 66 | 39 | 47 | 53 |
| Sex | Female | Female | Male | Male | Male |
| Ethnic origin | Chinese (han) | Chinese (han) | Chinese (han) | Chinese (han) | Chinese(han) |
| Place of residence | Huizhou,China | Yangjiang,China | Dongguan,China | Foshan,China | Foshan, China |
| Exposure to live poultry in previous 14 days | Yes | Yes | Yes | Yes | No |
| Underlying medical disorders | Diabetes | Duodenum ulcer | Hyperlipidaemia | No | [Hepatitis B](app:ds:hepatitis%20B) |
| Cigarette smoking | No  No | No | Yes | Yes | No |
