# Supplementary material for: Clinical, Virological and Immunological Features from Patients Infected with Re-Emergent Avian-Origin Human H7N9 Influenza Disease of Varying Severity in Guangdong Province
Source: PLoS One. 2015 Feb 27;10(2):e0117846. doi: 10.1371/journal.pone.0117846 (PMC4344233; doi:10.1371/journal.pone.0117846)
Supplement: S2 Table — (DOCX) [file pone.0117846.s004.docx]

S2 Table. The summary of the drugs given to the patients *

| Therapy | Patient 1 | Patient 2 | Patient 3 | Patient 4 | Patient 5 |
| --- | --- | --- | --- | --- | --- |
| Antiviral | Oseltamivir^♯^ (150mg, 9-22) | Oseltalmivir^♯^ (150mg,5-8) | Oseltamivir^♯^ (150mg,8-17) | Oseltamivir^♯^ (150mg, 11-22) | Oseltamivir^♯^(150mg, 4-21) |
|  |  | Peramivir^§^ (600mg,9-16) | Peramivir^§^ (600mg,18-22) |  |  |
|  |  | Zanamivir^♯^ (600mg,17-24) | Zanamivir^♯^ (600mg,23-32) |  |  |
| Antibiotic | Meropenem^♯^ (1g,11-22) | Vancomycin^♮^ (500mg,7-35) | Tienam^♮^ (1g,14-18) | Tienam^♮^ (1g,10-19) | Levofloxacin^§^ (0.5g,4-6) |
|  | Piperacillin tazobactam^♮^  (4.5g,23-27) | Tienam^♮^ (1g,7-11) | Vancomycin^§^ (500mg,14-31) | Azithromycin^§^ (0.5g,10-13) | Tienam^♮^ (1g,7-19) |
|  | Levofloxacin^§^ (0.5g,38-46) | Meropenem^♯^ (1g,12-21) | Sulperazone^♯^ (3g,20-23) | Moxifloxacin^§^ (400mg,14-16) | Moxifloxacin^§^ (400mg,12-19) |
|  |  | Zyvox^§^ (0.6g,25-38) | Ciprofloxacin^§^ (200mg,20-26) | Teicoplanin^§^ (400mg,17-27) | Vancomycin^§^ (500mg,7-8) |
|  |  | Levofloxacin^§^ (0.5g,36-43) |  | Sulperazone^♮^ (3g,20-29) |  |
| Antifungal | Voriconazole^§^ (200mg,14-37) | Diflucan^§^ (200mg,7-21) | Diflucan^§^ (200mg,14-15) | No | No |
|  |  | Voriconazole^§^ (200mg,22-30) | Cancidas^§^ (50mg,16-19) |  |  |
| Hormone^§^ | Methylprednisolone  (80mg, 10;40mg,12)  Synthetic insulin (6iu,10-12,14,19-23,25-27,29) | Methylprednisolone  (40mg,7,9) | Methylprednisolone  (80mg,14;40mg,15,20-25; 500mg,26-27;250mg,28-2) | No | No |
|  |  | Synthetic insulin (6iu,8) | Synthetic insulin  (6iu,15-16,18-19;40iu,24-30) |  |  |
|  |  |  | Noradrenaline  (2mg,16-18,22,25;4mg,21,23  8mg,24,26-28;16mg,29-3) |  |  |
| Immune globulin^§^ | Yes(10g,15,16,19) | Yes(10g,7,9) | Yes(10g,15,16,21,28,31) | No | No |
| Convalescent serum^§^ | No | No | Yes(20) | No | No |

* drug(dose, days after onset);§ quaque die; ♯ bis in die; ♮ ter in die.
