# Supplementary material for: Clinical, Virological and Immunological Features from Patients Infected with Re-Emergent Avian-Origin Human H7N9 Influenza Disease of Varying Severity in Guangdong Province
Source: PLoS One. 2015 Feb 27;10(2):e0117846. doi: 10.1371/journal.pone.0117846 (PMC4344233; doi:10.1371/journal.pone.0117846)
Supplement: S3 Table — (DOCX) [file pone.0117846.s005.docx]

S3 Table. Laboratory examination

|  | Normal range | Patient 1 | Patient 2 | Patient 3 | Patient 4 | Patient5 |
| --- | --- | --- | --- | --- | --- | --- |
| Total white cells (×10⁹ cells per L) | 4.0-10.0 | 3.78 | 22.26 | 31.52 | 3.62 | 3.9 |
| Total white cells<4 (×10⁹ cells per L) | — | Yes | No | No | Yes | Yes |
| [Neutrophil](app:ds:neutrophil) absolute value (×10⁹cells per L) | 1.8-8.0 | 1.2-2.8 | 0.75-21 | 1.3-20.4 | 2.31 | 3.1 |
| [Neutrophil](app:ds:neutrophil) ratio —(%) | 40-70 | 78.6 | 98.5 | 83.4 | 70.8 | 80.2 |
| Lymphocytes (×10⁹ cells per L) | 0·9-5.2 | 0.8 | 0.2 | 4.6 | 0.91 | 0.5 |
| Lymphocytopenia | — | Yes | Yes | No | No | Yes |
| Platelets (×10⁹ cells per L) | 100.0-400.0 | 278 | 180 | 139 | 110 | 83 |
| Thrombocytopenia | — | No | No | No | No | Yes |
| Haemoglobin (g/dL) | 110.1-160.0 | 99 | 72 | 83 | 148 | 164 |
| C-reactive protein>10 (mg/L) | 0-10.0 | 5.5 | 17.8 | 5.26 | — | 2.61 |
| Procalcitonin>0.5 ng/ml | 0-0.05 | Yes | 1.14 | 1.14 | 0.37 | 0.49 |
| Aspartate aminotransferase>40 (U/L) | 5.0-40.0 | 57 | 65.2 | 64.8 | 57.5 | 95.1 |
| Creatinine>133 (μmol/L) (1.5mg/dl) | 44.0-133.0 | 44 | 30 | 212 | 65-102.3 | 109 |
| Lactate dehydrogenase >250(U/L) | 109.0-250.0 | 328 | 360 | 2069 | 403.2 | 493 |
| Creatine kinase >200 U/liter | 10.0-190.0 | 54 | 799 | 2565 | 540 | 2177 |
| Myoglobulin>80 μg/ml | 0-70 | 37 | 155.7 | 250.3 | 176.0 | 164 |
| Potassium — mmol/liter | 3.50-5.30 | 3.85 | 3.23-4.23 | 4.76-5.38 | 3.33 | 3.4 |
| Sodium — mmol/liter | 134.0-145.0 | 144.7 | 131-138.3 | 144-148.6 | 127.2 | 126.9 |
| d -dimer >0.5 mg/liter | — | Yes | Yes | Yes | No | 1699 |
| Chest radiologic findings |  |  |  |  |  |  |
| Involvement of both lungs | — | Yes | Yes | Yes | Yes | Yes |
| Ground-glass opacity | — | Yes | Yes | Yes | Yes | Yes |
| Consolidation | — | Yes | Yes | Yes | Yes | Yes |
